# Preoperative tumor size is associated with deep myometrial invasion and lymph node metastases and is a negative prognostic indicator for patients with endometrial carcinoma

**DOI:** 10.18632/oncotarget.25248

**Published:** 2018-05-01

**Authors:** Kohei Nakamura, Kentaro Nakayama, Noriyoshi Ishikawa, Toshiko Minamoto, Tomoka Ishibashi, Kaori Ohnishi, Hitomi Yamashita, Ruriko Ono, Hiroki Sasamori, Sultana Razia, Mohammad Mahmud Hossain, Shanta Kamrunnahar, Masako Ishikawa, Satoru Kyo

**Affiliations:** ^1^ Department of Obstetrics and Gynecology, Shimane University School of Medicine, Izumo 6938501, Japan; ^2^ Department of Organ Pathology, Shimane University School of Medicine, Izumo 6938501, Japan

**Keywords:** endometrial carcinoma, tumor size, overall survival, progression-free survival, myometrial invasion

## Abstract

We examined the usefulness of evaluating tumor size determined using preoperative magnetic resonance imaging (MRI) for prognosis in patients with endometrial carcinoma (EC). Patients (*N* = 184) with EC who underwent surgery at Shimane University Hospital between 1997 and 2013 were enrolled. We investigated the association between the tumor size of EC assessed prior to surgery by MRI (anteroposterior [AP], transverse [TV], and craniocaudal [CC] diameters) and various clinical parameters including deep myometrial invasion and lymph node metastases. We subsequently examined the prognostic significance of tumor size in patients with EC. Survival analysis was performed using the Kaplan-Meier method, and prognostic factors were evaluated using the Cox’s proportional hazards regression model.

Multivariate analysis identified increased AP diameter as an independent negative prognostic factor for overall survival (OS) (*P* = 0.037). A long AP diameter has prognostic value and the potential to be a predictive marker for surgical outcomes in patients with EC. Furthermore, AP diameter exhibited the greatest area under the curve (AUC) (0.727) for deep myometrial invasion, and CC diameter had the greatest AUC for lymph node metastases (0.854). Evaluation of tumor size parameters may aid in the identification of high-risk populations, which could improve treatment selection and patient outcomes.

## INTRODUCTION

Endometrial cancer (EC) is the most common gynecologic malignancy, with an annual incidence of 320,000 and a mortality rate of 76,000 deaths per year worldwide [1, 2]. The International Federation of Gynecology and Obstetrics (FIGO) staging system is used by clinicians for prognostication and to guide surgical management. Interestingly, patients with the same disease stage may experience very different clinical courses [1, 3]. To understand the reasons for these differences, many investigators have evaluated the influence of various tumor attributes, such as histological subtype and FIGO stage, grade, depth of myometrial invasion, and vascular invasion on prognosis [3–5]. Unfortunately, preoperative evaluations generally require invasive, costly, and time-consuming procedures such as fractional curettage or hysteroscopic assessment [1, 6–8]. Early detection and improvements in surgical techniques and chemotherapies have contributed to better prognoses. However, precise predictions of prognosis remain a challenge despite being necessary for guiding clinical decision-making regarding optimal treatment.

Previous studies on cervical, breast, and renal cancers have demonstrated that increased tumor size is associated with a poorer prognosis [9–12]. In their study, Gusberg et al. [13] reported that tumor size was an indicator of prognosis in EC and used uterine size as a surrogate marker for tumor size [13]. Many studies have since reported conflicting results when examining the usefulness of tumor size as a prognostic indicator. Several studies have demonstrated that tumor size is significantly associated with lymph node metastases and survival [14–16], leading them to recommend the addition of tumor size to routine assessments for identifying low-risk endometrial cancer patients [17]. Other studies, however, have challenged such a correlation [18, 19]. Moreover, the FIGO staging system does not mention tumor size for endometrial cancer diagnosis [20]. Soliman et al. [21] outlined factors used to guide surgical decision-making for patients with endometrial cancer and focused on the indications for lymphadenectomy; interestingly, the authors did not consider tumor size as part of their survey [21].

The advantages of evaluating tumor size include ease of evaluation and the lack of a need for extra resources or a skilled pathologist. In contrast, intraoperative assessment of other prognostic indicators such as grade, myometrial invasion, and lymph metastases require well-trained pathologists and additional resources, which can vary depending on whether frozen or paraffin sections are required [22, 23]. Magnetic resonance imaging (MRI) is another modality that can be used to assess tumor size; however, to our knowledge, studies examining the reproducibility of tumor size measurements by MRI are lacking. Moreover, there is currently no established optimal threshold value for risk assessment based on tumor size.

Here, the primary aim was to examine the relationship between preoperative tumor size measured by MRI and staging variables including deep myometrial invasion and lymph node metastases among patients with EC. The secondary aim was to clarify the prognostic significance of tumor size in patients with EC.

## RESULTS

### Patient and clinical features

A total of 184 patients with EC were enrolled. Their clinical and pathological characteristics are shown in Table 1. Regarding histology, 87.5% (161/184) of tumors were endometrioid, 8.1% (15/184) were serous, and 4.3% (8/184) were clear cell. In our analysis, we divided the histology into two categories (endometrioid vs. others).

**Table 1:** Clinical characteristics of the patient population (*n* = 184)

Characteristic	No. of patients	%
Age at diagnosis, y		
< 60	93	51
≥ 60	91	49
FIGO stage		
I, II	143	78
III, IV	41	22
Histology		
Endometrioid	161	88
Other	23	12
Grade		
G1	85	46
G2, G3	99	54
Myometrial invasion		
< 1/2	119	65
≥ 59/	65	35
Lymph metastasis		
No	136	74
Yes	19	10
Not assessed	29	16
Venous invasion		
No	125	68
Yes	59	32
Lymphatic invasion		
No	104	
Yes	80	
AP diameter, mm		
< 52	146	
≥ 46	38	
TV diameter, mm		
< 37	119	
≥ 19	65	
CC diameter, mm		
< 28	111	
≥ 11	73	

FIGO, International Federation of Gynecology and Obstetrics; AP, anteroposterior; TV, transverse; CC, craniocaudal.

### Relationships between tumor size parameters and prognosis in patients with EC and selection of the optimal threshold value for tumor size

**Figure 1:**
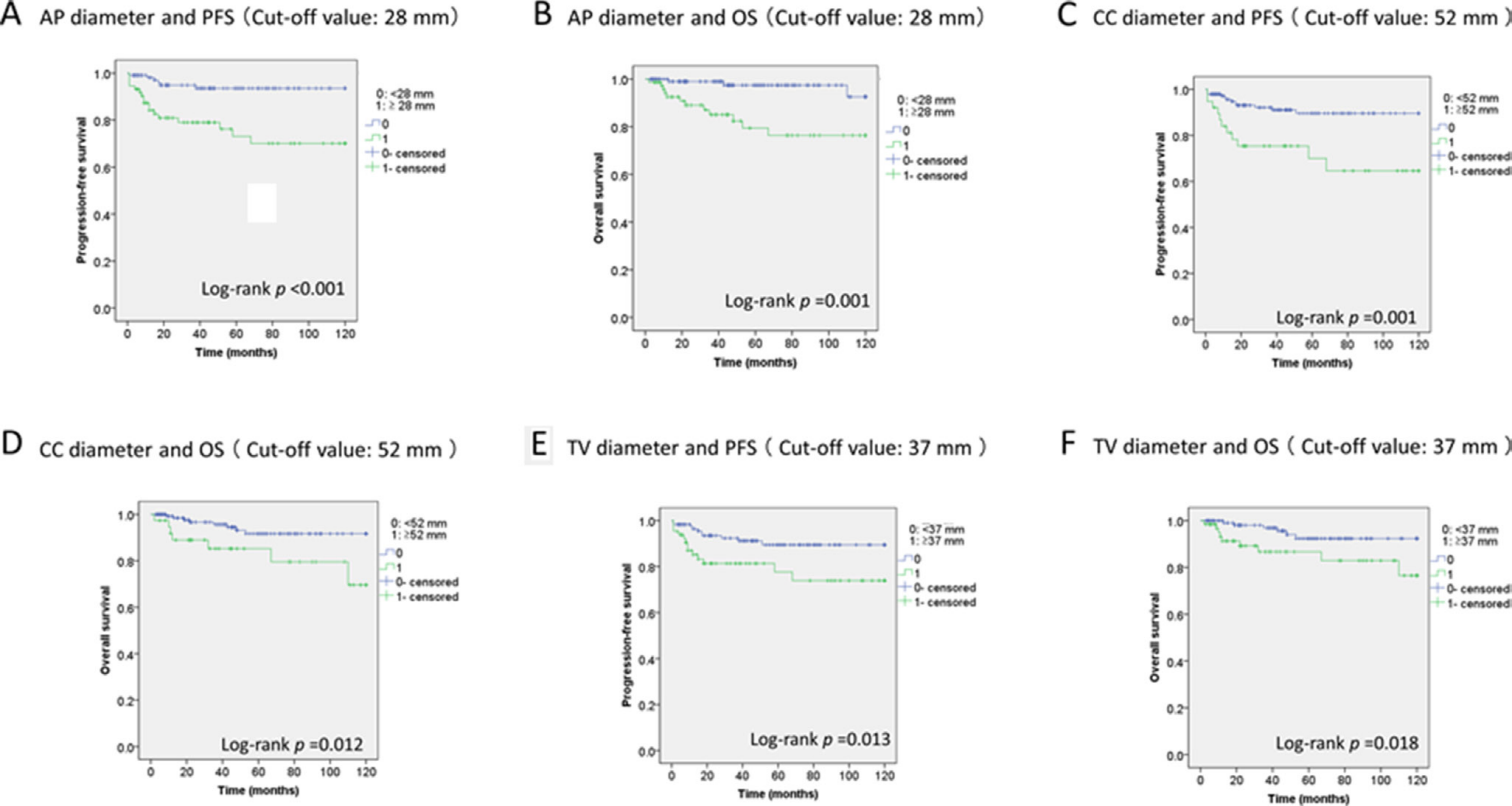
Association of the three tumor size measurements and prognosis Kaplan-Meier estimates of the usefulness of the AP diameter in the prognosis of PFS (**A**) and OS (**B**), the CC diameter in the prognosis of PFS (**C**) and OS (**D**), and the TV diameter in the prognosis of PFS (**E**) and OS (**F**) in patients with endometrial carcinoma.

We used receiver operating characteristic (ROC) analysis to define the optimal threshold value for each tumor size measurement in the prediction of progression-free survival (PFS) and overall survival (OS). Tumor size measurements along each of 3 orthogonal planes (anteroposterior [AP], transverse [TV], and craniocaudal [CC]) were associated with PFS and OS in Kaplan-Meier analysis. The area under the curve (AUC) for the AP diameter was 0.719 for PFS, and the threshold value was 28 mm (Supplementary Figure 1A); the AP diameter was significantly associated with PFS (*P* < 0.001) (Figure 1A). The AUC for the AP diameter was 0.746 for OS, and the threshold value was 28 mm (Supplementary Figure 1B); the AP diameter was significantly associated with OS (*P* = 0.001) (Figure 1B). The AUC for the CC diameter was 0.663 for PFS, and the threshold value was 52 mm (Supplementary Figure 2A); the CC diameter was significantly associated with PFS (*P* = 0.001) (Figure 1C). The AUC for the CC diameter was 0.660 for OS, and the threshold value was 52 mm (Supplementary Figure 2B); the CC diameter was significantly associated with OS (*P* = 0.012) (Figure 1D). The AUC for the TV diameter was 0.603 for PFS, and the threshold value was 37 mm (Supplementary Figure 3A); the TV diameter was significantly associated with PFS (*P* = 0.013) (Figure 1E). The AUC for the TV diameter was 0.596 for OS, and the threshold value was 37 mm (Supplementary Figure 3B); the TV diameter was significantly associated with OS (*P* = 0.018) (Figure 1F).

### Univariate and multivariate analyses of prognostic factors in patients with EC

The association between age, clinical stage, histological type, grade, deep myometrial invasion, lymph node metastasis, venous invasion, lymphatic invasion, and the three tumor size measurements and survival were investigated using univariate analyses. For PFS, stage (*P* < 0.001), histologic type (*P* = 0.015), grade (*P* = 0.003), deep myometrial invasion (*P* = 0.012), lymph node metastasis (*P* < 0.001), venous invasion (P = 0.002), lymphatic invasion (*P* < 0.001), AP diameter (*P* = 0.001), CC diameter (*P* = 0.002), and TV diameter (*P* = 0.017) were significant predictors. Multivariate analysis revealed that advanced stage (III/IV) (hazard ratio [HR] = 4.441; 95% confidence interval [CI] = 1.463–13.476; *P* = 0.008) was an independent negative predictor of PFS (Table 2). For OS, as shown in Table 3, stage (*P* < 0.001), histologic type (*P* = 0.027), grade (*P* = 0.033), lymph node metastasis (*P* = 0.001), venous invasion (P = 0.017), lymphatic invasion (*P* = 0.005), CC diameter (*P* < 0.001), AP diameter (*P* = 0.012), and TV diameter (*P* = 0.018) were significant predictors. Multivariate analysis showed that advanced stage (stage III/IV) (HR, 5.756; 95% CI, 1.708–19.397; *P* = 0.005) and AP diameter (HR, 5.285; 95% CI, 1.110–25.170; *P* = 0.037) were significant independent negative predictors of OS (Table 3).

**Table 2:** Univariate and multivariate analyses of prognostic factors for progression-free survival

Factor	Univariate Analysis			Multivariate Analysis		
	Hazard Ratio	95% CI	*P*	Hazard Ratio	95% CI	*P*
Age at diagnosis, y	2.379	0.978–5.784	0.056	-	-	-
FIGO stage	9.705	3.795–24.816	< 0.001	4.441	1.463–13.476	0.008
Histology	3.203	1.256–8.171	0.015	-	-	-
Grade	8.951	2.098–38.177	0.003	3.193	0.680–14.980	0.141
Myometrial invasion	2.957	1.264–6.921	0.012	-	-	-
Lymph metastasis	10.197	3.921–26.518	< 0.001	-	-	-
Venous invasion	3.726	1.589–8.734	0.002	-	-	-
Lymphatic invasion	7.124	2.405–21.106	< 0.001	2.770	0.795–9.649	0.110
AP diameter	4.737	1.867–12.020	0.001	-	-	-
CC diameter	3.702	1.633–8.392	0.002	-	-	-
TV diameter	2.729	1.195–6.230	0.017	-	-	-

AP, anteroposterior; TV, transverse; CC, craniocaudal; CI, confidence interval; FIGO, International Federation of Gynecology and Obstetrics; N/A, not available.

**Table 3:** Univariate and multivariate analyses of overall prognostic factors

Factor	Univariate Analysis			Multivariate Analysis		
	Hazard Ratio	95% CI	*P*	Hazard Ratio	95% CI	*P*
Age at diagnosis, y	1.548	0.551–4.352	0.407	-	-	-
FIGO stage	9.642	3.067–30.311	< 0.001	5.756	1.708–19.397	0.005
Histology	3.699	1.164–11.755	0.027	-	-	-
Grade	5.049	1.138–22.407	0.033	-	-	-
Myometrial invasion	2.650	0.918–7.644	0.071	-	-	-
Lymph metastasis	2.529	1.474–4.339	0.001	-	-	-
Venous invasion	3.658	1.264–10.589	0.017	-	-	-
Lymphatic invasion	6.405	1.774–23.120	0.005	-	-	-
AP diameter	6.489	1.830–23.013	< 0.001	5.285	1.110–25.170	0.037
CC diameter	3.388	1.228–9.347	0.012	-	-	-
TV diameter	3.256	1.157–9.165	0.018	-	-	-

AP, anteroposterior; TV, transverse; CC, craniocaudal; CI, confidence interval; FIGO, International Federation of Gynecology and Obstetrics; N/A, not available.

### Correlation between the three tumor size measurements and deep myometrial invasion in patients with EC

**Figure 2:**
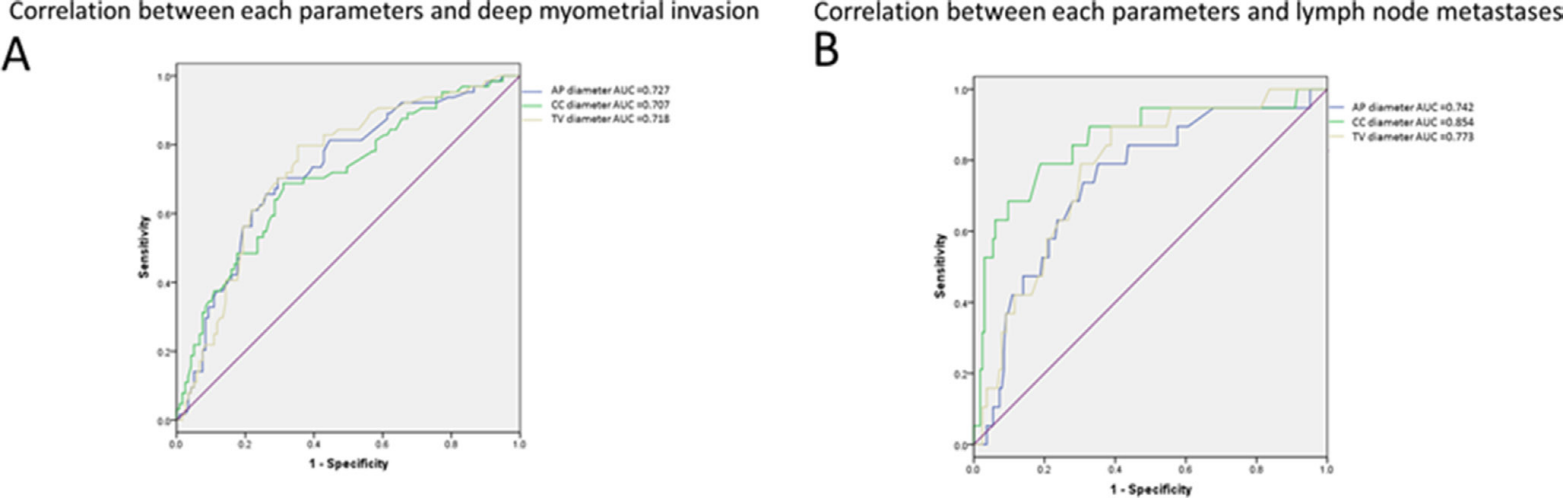
Characteristics for identifying deep endometrial invasion and lymph node metastases Receiver operating characteristic curves for the three tumor size measurements for identifying deep myometrial invasion (**A**) and lymph nodemetastases (**B**) in patients with endometrial carcinoma.

Using the above threshold values for each tumor size measurement, ROC curves indicating the effectiveness of the diameter measurements for predicting deep myometrial invasion (Figure 2A) and lymph node metastases (Figure 2B) demonstrated that the AP diameter had the greatest AUC (0.727) for deep myometrial invasion, while the CC diameter had the greatest AUC for lymph node metastases (0.854).

## DISCUSSION

To our knowledge, this is the first cohort study to demonstrate that tumor size (in particular, the AP tumor diameter) is a robust indicator of prognosis in patients with EC. ROC curve analysis revealed that the optimal thresholds for predicting prognosis were AP diameter > 28 mm, CC diameter > 52 mm, and TV diameter > 37 mm. Furthermore, we showed that tumor size is significantly associated with deep myometrial invasion (especially AP diameter, threshold value: 28 mm), and lymph node metastases (especially CC diameter, threshold value: 52 mm). While the prognosis of EC is typically favorable [24], it is poor in some patients. The association between tumor size and prognosis and other clinical prognostic indicators has garnered increasing attention recently. In previous studies, the probability of myometrial invasion increases with the tumor size [14, 15]. There are two reasons for this. First, when the tumor is growing, it invades the myometrium because there is limited space in the uterine cavity. Second, when the tumor invades the myometrium, the cancer cells first invade the lymphatic or vascular system and next invade the lymph nodes. Previous studies have demonstrated that deep myometrial invasion correlates with surgical factors such as lymph node metastases and distant metastases in EC [25]. However, accurate assessment of myometrial invasion is difficult to obtain using only MRI. For example, it is not uncommon to find that cancers that are classified as stage IA preoperatively turn out to be stage IB postoperatively. If the preoperative diagnosis is uncertain, then the surgical method may be inadequate. For example, whereas pelvic lymphadenectomy is appropriate for stage IB cases (deep myometrial invasion), this should be omitted in patients with a preoperative diagnosis of stage IA and G1. Therefore, it is essential to identify novel factors that are associated with deep myometrial invasion and can be evaluated preoperatively. Tumor size is a potentially useful marker for this purpose that can be obtained with relatively little effort and low interobserver variability.

The same is true of lymph node metastasis. Enlarged lymph nodes seen on preoperative MRI are not always found to be the result of metastasis postoperatively. Several surgical or pathological risk factors for predicting deep myometrial invasion or lymph node metastases have been reported for EC including histologic type, grade, and tumor extent [26–29]; one model used tumor size greater than 2 cm according to gross examination of hysterectomy tissue as a predictive factor [26]. Limitations of these models include reliance on surgical and pathological outcomes that cannot be obtained preoperatively. However, direct comparison of macroscopic tumor diameter measurements between fresh tissue and preoperative MRI is difficult because of differences in the planes of the section that can be obtained for tumor measurements and possible distortion of tumor tissue *in vivo* compared to *ex vivo*. As a result, the optimal tumor size threshold values may not be transferable from *in vivo* MRI-based to *ex vivo* gross section-based measurements. Despite this, both *in vivo* and *ex vivo* studies consistently provide evidence of the metastatic potential and negative prognostic impact of large tumor size in EC [26]. Sigmund et al. [29] demonstrated that AP tumor diameter > 2 cm and CC tumor diameter > 4 cm as measured using MRI were associated with prognosis; these results are similar to the findings of the present study [29].

The significance of tumor size as an indicator of pelvic lymphadenectomy in patients with low-risk EC should also be considered. At present, unlike its diagnostic significance, the therapeutic significance of pelvic lymphadenectomy in low-risk EC remains unclear. Previous reports have demonstrated that pelvic lymphadenectomy may be associated with good prognosis [30–34]; however, these studies have not all been randomized controlled studies. Recently, two randomized controlled studies demonstrated that pelvic lymphadenectomy was not associated with prognosis in patients with low-risk EC [35, 36]. However, in most cases, excluding clearly low-risk cases, lymphadenectomy is considered. Our results indicate that omitting lymphadenectomy in patients with presumed stage I disease with long AP or CC tumor diameter is associated with a risk of recurrence and is therefore not clinically indicated. The results of the present study may be helpful for guiding clinical decision-making regarding lymphadenectomy.

We previously reported that loss of MMR protein expression was identified in 42 of 149 (28.2%) patients with endometrial cancer, and microsatellite instability is a biomarker for immune checkpoint inhibitors in endometrioid endometrial cancer [37]. In this study, we analyzed the relationship between tumor size and prognosis in patients with MMR deficiency (*N* = 42). However, there were no significant differences among them (data not shown). MMR deficient endometrioid carcinoma may behave in a different way in terms of tumor size, but we think that the small sample size may affect this result. Therefore, we must investigate the tumor size significance in a large population of patients with MMR deficiency in the future. Furthermore, other preoperative biomarkers such as p53, hormone receptor, and DNA ploidy status in preoperative biopsies should also be evaluated in the future.

This study has some limitations. First, this was a retrospective study, and therefore, we could not control for all sources of bias. Second, interobserver variability was not evaluated in this study. Third, the tumor growth pattern was not considered in this study. There are various tumor growth patterns such as a surface spreading pattern, an endomyometrium invading pattern, and a polypoid pattern. In previous studies, the influence of the tumor growth pattern on the prognosis was not discussed. We should investigate the influence of tumor growth patterns on the prognosis of EC in the future.

Our findings indicate that tumor size parameters, especially AP diameter, evaluated preoperatively using MRI, are predictive of the presence of deep myometrial invasion and lymph node metastases.

In conclusion, we identified increased AP diameter as a predictive factor of surgical outcomes in patients with EC. Furthermore, tumor size measured preoperatively using MRI is predictive of deep myometrial invasion and lymph node metastases. Based on our findings, preoperative tumor measurements made using MRI may constitute clinically useful markers to assess recurrence risk and guide tailored surgical treatment in EC.

## MATERIALS AND METHODS

### Subjects

This study was approved by the Ethics Committee of Shimane Medical University. We investigated 184 patients with EC who underwent surgery at the University Hospital of Shimane between 1997 and 2016. Patients with insufficient data, nonsurgical treatment, secondary malignancies, hematologic diseases, and those who lacked MRI data were excluded.

Diagnoses were made according to conventional morphological examinations of hematoxylin and eosin-stained sections, and tumors were classified according to the World Health Organization classification system. The FIGO classification system was used for tumor staging and grading. All patients underwent surgery (total abdominal hysterectomy and bilateral salpingo-oophorectomy), and those with cancers greater than stage 1a and grade 1 underwent pelvic lymph node dissection and were administered adjuvant platinum and taxane chemotherapy. Patients with stage 1a and grade 1 EC did not undergo pelvic lymph node dissection or receive adjuvant chemotherapy.

### Measurement of tumor size

Preoperative MRI was performed 60 days before surgery in all cases. All MRI images were reviewed by two independent radiologists who were blinded to patient data and who conducted assessments for tumor size and histological diagnosis.

**Figure 3:**
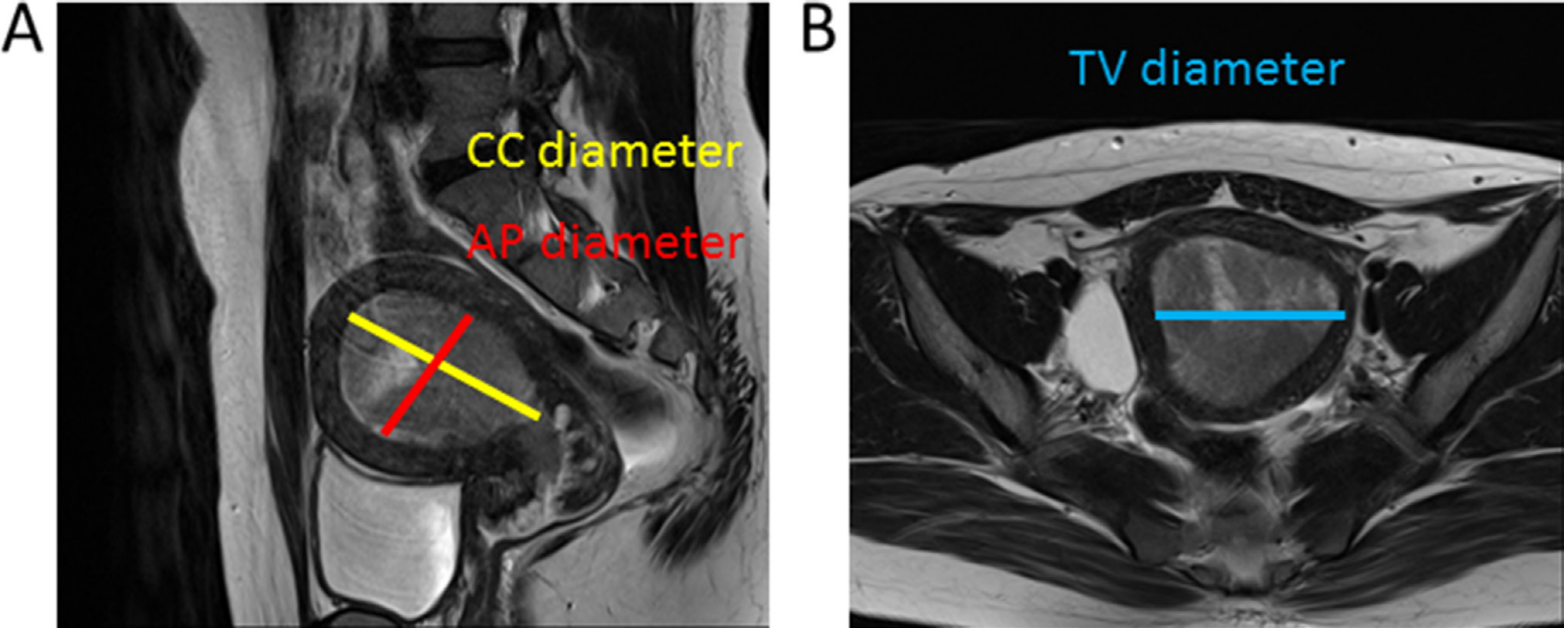
Images used to determine tumor diameter The maximum tumor diameters were determined along three orthogonal planes. AP and maximum CC diameter were determined using the sagittal oblique T2-weighted image (**A**), and the TV diameter was determined using the coronal image (**B**).

The radiologists recorded their assessments of MRI images on a standardized form. Tumor size measurements were made along 3 orthogonal planes: AP and TV diameters were measured using axial contrast-enhanced T1-weighted oblique images (at right angles to the long axis of the uterus), and the CC diameter was determined using sagittal T2-weighted images (Figure 3A, 3B).

### Selection of threshold value

ROC curve analysis was conducted to establish the threshold value for all tumor size measurements required for diagnosing deep myometrial invasion and lymph node metastases. The sensitivity and specificity of each outcome were plotted to generate ROC curves. The threshold value was selected as the value that was closest to the point with both maximum sensitivity and specificity.

### Statistical analysis

Statistical analyses were carried out using SPSS software (version 19.0 for Windows; IBM Corp., Armonk, NY, USA). ROC curve analysis was applied to determine the threshold value for each tumor size measurement. We used binomial logistic regression analysis for univariate analysis for ordered categorical variables. We used the following clinical factors for modeling: patient age at diagnosis (< 60 vs. ≥ 60 years), stage (I/II vs. III/IV), histologic type (endometrioid vs. others), tumor grade (1 vs. 2/3), myometrial invasion (< 1/2 vs. ≥ 1/2), lymph metastasis, venous invasion, lymphatic invasion, and three tumor size measurements (AP, TV, and CC diameter).

ROC analysis was used to determine the usefulness of the three tumor size measurements for the diagnosis of deep myometrial invasion and lymph node metastases. The optimal threshold values (rounded to the nearest centimeter) were chosen as those producing the best separation of the Youden index between the groups.

PFS and OS were the endpoints of the analysis. PFS indicates the time between initial diagnosis and initial recurrence of the disease. Patients with no recurrence at their most recent follow-up were censored at the follow-up. OS indicates the time between initial diagnosis and death. Patients who were living at their most recent follow-up were censored at the follow-up. Kaplan-Meier curves and log-rank tests were used to plot the survival data and determine the statistical significance of survival differences. We entered variables that were significant (*P* < 0.05) in the univariate analysis into the multivariate analysis. Cox’s proportional hazards model was used for prognostic analysis. Data of patients who were lost to follow-up were censored. All reported *P* values were two-sided, and a *P* value < 0.05 was considered significant.

## SUPPLEMENTARY MATERIALS FIGURES



## Abbreviations

AP: anteroposterior
AUC: area under the curve
CC: craniocaudal
EC: endometrial carcinoma
FIGO: The International Federation of Gynecology and Obstetrics
MRI: magnetic resonance imaging
OS: overall survival
PFS: progression-free survival
ROC: receiver operating characteristic
TV: transverse
