# Supplementary material for: Preoperative tumor size is associated with deep myometrial invasion and lymph node metastases and is a negative prognostic indicator for patients with endometrial carcinoma
Source: Oncotarget. 2018 May 1;9(33):23164–72. doi: 10.18632/oncotarget.25248 (PMC5955431; doi:10.18632/oncotarget.25248)
Supplement: Supplementary file 1 [file oncotarget-09-23164-s001.pdf]

# Preoperative tumor size is associated with deep myometrial invasion and lymph node metastases and is a negative prognostic indicator for patients with endometrial carcinoma

## SUPPLEMENTARY MATERIALS

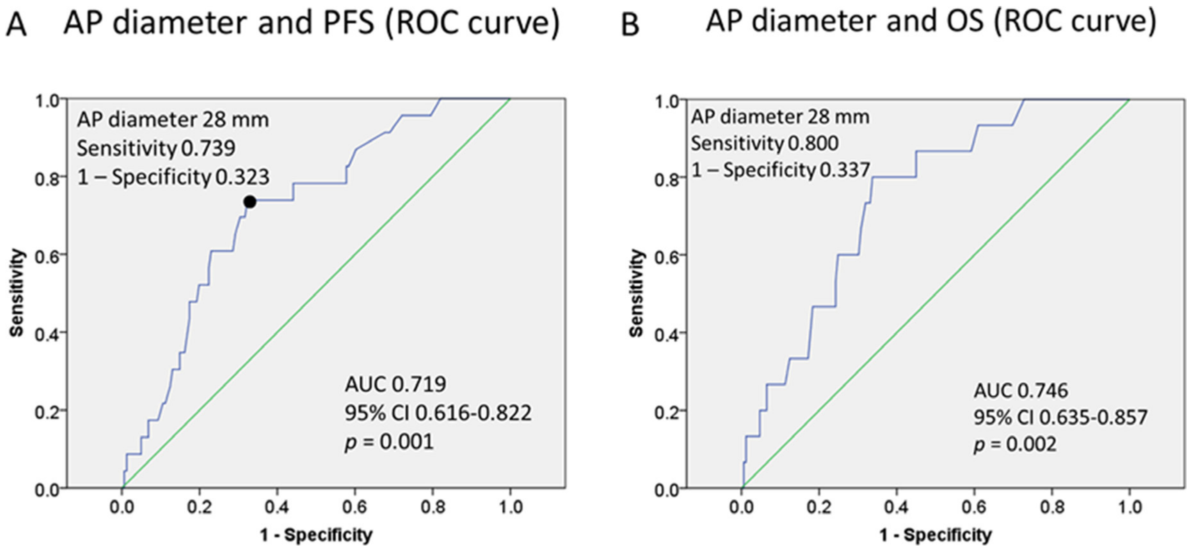


**Supplementary Figure 1:** The ROC analysis of AP diameter in the prognosis of PFS (**A**) and OS (**B**).

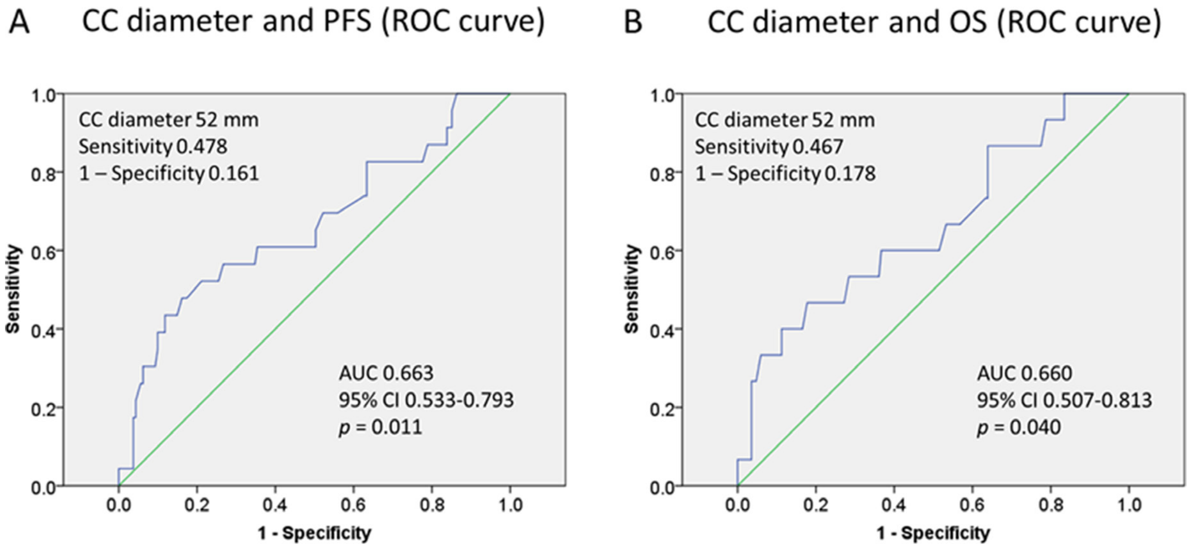


**Supplementary Figure 2:** The ROC analysis of CC diameter in the prognosis of PFS (**A**) and OS (**B**).

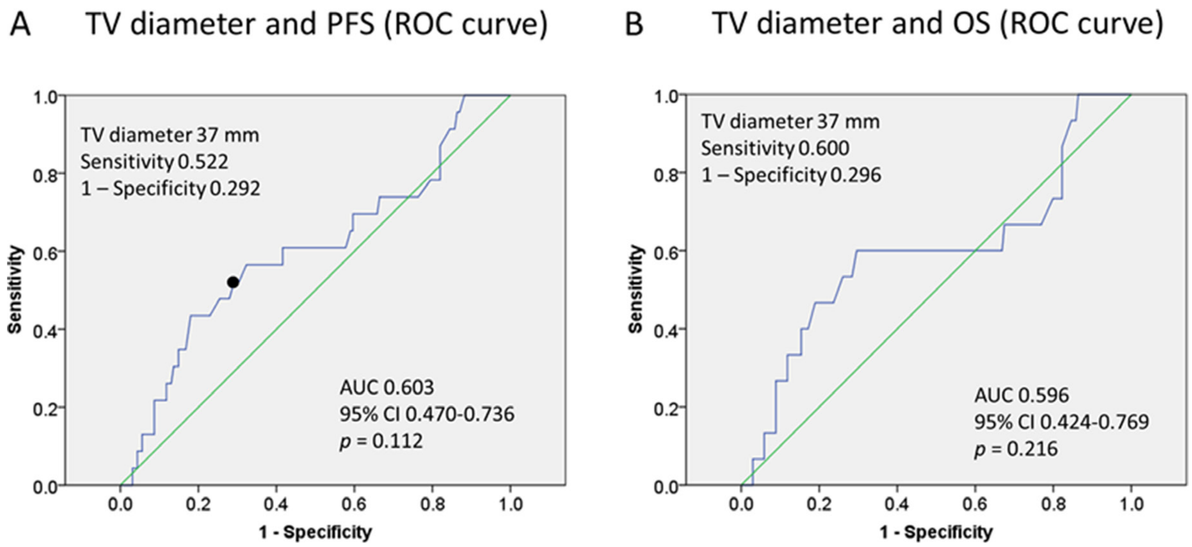


**Supplementary Figure 3:** The ROC analysis of TV diameter in the prognosis of PFS (**A**) and OS (**B**).
